# Tissue-Specific Delivery of CRISPR Therapeutics: Strategies and Mechanisms of Non-Viral Vectors

**DOI:** 10.3390/ijms21197353

**Published:** 2020-10-05

**Authors:** Karim Shalaby, Mustapha Aouida, Omar El-Agnaf

**Affiliations:** 1 Division of Biological and Biomedical Sciences (BBS), College of Health & Life Sciences (CHLS), Hamad Bin Khalifa University (HBKU), Doha 34110, Qatar; kshalaby@hbku.edu.qa; 2 Neurological Disorders Research Center, Qatar Biomedical Research Institute (QBRI), Hamad Bin Khalifa University (HBKU), Doha 34110, Qatar

**Keywords:** CRISPR-Cas, gene editing, gene therapy, non-viral vectors, cell-penetrating peptides

## Abstract

The Clustered Regularly Interspaced Short Palindromic Repeats (CRISPR) genome editing system has been the focus of intense research in the last decade due to its superior ability to desirably target and edit DNA sequences. The applicability of the CRISPR-Cas system to in vivo genome editing has acquired substantial credit for a future in vivo gene-based therapeutic. Challenges such as targeting the wrong tissue, undesirable genetic mutations, or immunogenic responses, need to be tackled before CRISPR-Cas systems can be translated for clinical use. Hence, there is an evident gap in the field for a strategy to enhance the specificity of delivery of CRISPR-Cas gene editing systems for in vivo applications. Current approaches using viral vectors do not address these main challenges and, therefore, strategies to develop non-viral delivery systems are being explored. Peptide-based systems represent an attractive approach to developing gene-based therapeutics due to their specificity of targeting, scale-up potential, lack of an immunogenic response and resistance to proteolysis. In this review, we discuss the most recent efforts towards novel non-viral delivery systems, focusing on strategies and mechanisms of peptide-based delivery systems, that can specifically deliver CRISPR components to different cell types for therapeutic and research purposes.

## 1. Introduction

The CRISPR genome editing system was first identified as short repeats of DNA downstream of the *iap* gene of *Escherichia coli* [1]. In 2002, it was referred to as “CRISPR”, and the CRISPR-associated genes were discovered as highly conserved gene clusters located adjacent to the repeats [2]. A series of studies later revealed that CRISPR is a bacterial immune system that dismantles invading viral genetic material and integrates short segments of it within the array of repeated elements [3,4]. This array is transcribed and the transcript is cut into short CRISPR RNAs (crRNAs) each carrying a repeated element together with a spacer consisting of the viral DNA segment [5]. This crRNA guides a CRISPR-associated (Cas) nuclease towards invading viral DNA to cleave it and inactivate viral infection at the next occurrence [5]. Starting 2013, the fusion of a crRNA containing a guiding sequence with a trans-activating RNA (tracrRNA) bearing the repeat, into a single-guide RNA (sgRNA) has opened the doors for gene editing in mammalian cell lines and many other species [6,7,8,9,10].

**Figure 1:**
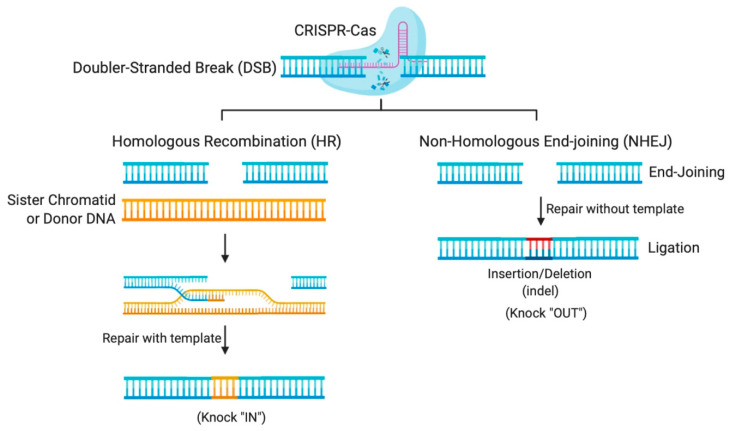
Gene editing by Clustered Regularly Interspaced Short Palindromic Repeats (CRISPR)-Cas systems relies on DNA repair pathways. DNA double-stranded breaks (DSBs) are repaired in cells via the error-prone non-homologous end-joining (NHEJ), or the error-free homologous recombination (HR), the most common form of homology-directed repair (HDR). The DSB repair through NHEJ creates small insertions or deletions (indels), while HDR requires a repair template, which could be a sister chromatid, another homologous region, or an exogenous repair donor.

The flexibility of the CRISPR system lies in its programmable Cas nuclease, which utilizes a guide RNA (gRNA) sequence to reach the desired complementary genomic sequence [11]. CRISPR-Cas systems open venues for applications in genetic functional screening, disease modeling, and gene modification [12]. The double-stranded breaks (DSBs) created by the Cas nuclease makes deletions or insertions at precise genomic loci possible [12]. In nature, DSBs can occur randomly during replication or due to environmental factors and are repaired in our cells using the homologous DNA copy as a template in a natural DSB DNA repair process called homology-directed repair (HDR) [13]. This cellular DNA repair pathway can thus be employed to copy a co-introduced artificial DNA template (donor) carrying a desired sequence into the target cleavage site (Figure 1). However, HDR is only activated when the homologous sister chromatid is available, usually in S and G2 phases, during the cell cycle [13]. Otherwise, HDR is suppressed and DSB repair is maintained through a distinct repair pathway called non-homologous end-joining (NHEJ) [13]. In non-dividing cells, such as neurons, gene editing is mostly carried out using NHEJ, where broken DNA ends are directly ligated without any requirement of sequence homology [13]. NHEJ mediated repair, however, is error-prone and tends to produce arbitrary mutations at the site of ligation of DSBs. Often, these mutations create premature stop codons or frame-shifts that are capable of knocking-out the gene of interest, thus providing means to investigate gene function, develop a disease model, or to cease the expression of a harmful mutant protein for therapy [12].

**Figure 2:**
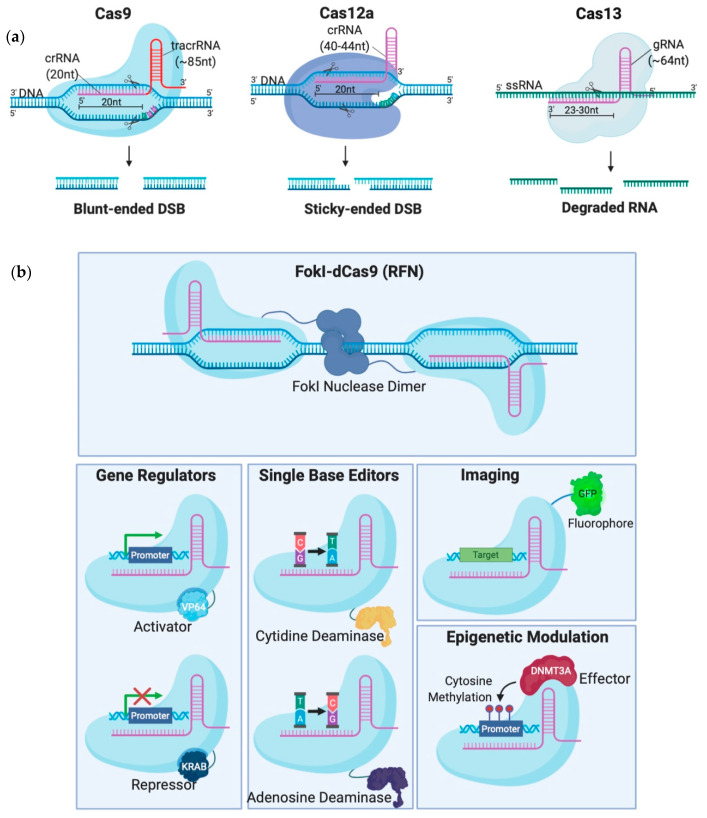
Different Clustered Regularly Interspaced Short Pallindromic Repeats (CRISPR)-Cas systems and deficient CRISPR-associated (dCas) platforms used in gene editing and other types of applications. (**a**) CRISPR-Cas9, 12a and 13 are used mainly in gene editing to target DNA and RNA, respectively. Each platform is composed of two components: the endonuclease sub-unit and single guide RNA (sgRNA). (**b**) Various CRISPR–dCas-fused platforms used in gene editing such as RNA-guided *Flavobacterium okeanokoites* (FokI) nuclease (RFN), Base editing (cytidine deaminases and adenosine deaminases), Gene regulators (transcriptional activators such as VP46, transcriptional repressors such as KRAB), imaging (fluorescent protein tags such as GFP) and epigenetic modulation (transcriptional modulators such as DNMT3A for cytosine methylation).

Although CRISPR is not the first site-specific nuclease used in gene-editing, it is the simplest, most rapid, and cheapest of the designer nucleases. CRISPR-Cas systems are classified into 2 classes, 6 types and 33 subtypes, differing in their genes, protein sub-units and the structure of their gRNAs [14]. Class 2 systems have been the most preferred for genetic manipulation, due to their simple design solely involving a sequence-specific monomeric Cas nuclease guided by a variable gRNA molecule [15]. DSBs are created by the Cas nuclease after gRNA aligns with the target DNA sequence via base pair (bp) matching [16]. Thus, only simple modification of the gRNA sequence is enough to target different locations in the genome [12]. The availability of target sites is limited due to the requirement of a protospacer adjacent motif (PAM) sequence adjacent to the target sequence [16]. But PAMs are highly common and virtually any site in the genome can be targeted [17]. Class 2 CRISPR systems which have been commonly employed for genome editing in mammalian cells with reportedly high efficiency and specificity are summarized in Table 1 and illustrated in Figure 2A [6,15,17,18,19,20,21,22,23,24,25,26,27,28,29].

To avoid inherent problems associated with the formation of DNA DSBs in cells, researchers have fused catalytically deficient Cas9 (dCas9) to single-base editors that can convert single nucleotides without the need to create a DSB which is very attractive for the development of gene therapy [30,31]. Fusion of dCas9 with other proteins can also serve as regulators of gene expression [32,33,34,35], epigenetic histone re-modulators [36], or fluorescent reporters for genome labelling [37] (Figure 2B).

## 2. Potential and Application of CRISPR Therapeutics

CRISPR-Cas systems discussed above have the ability to be used for the development of corrective gene therapeutics for a huge number of genetic mutations contributing to disease [38,39,40]. In only a few years after its discovery, CRISPR had already transformed the field of biomedical research, with the first Food and Drug Administration-approved ex vivo clinical trial started in 2018 [41] and the first in vivo clinical trial in 2019 [42] along with numerous clinical trials under way as of today. CRISPR therapies being tested in clinical trials launched by Vertex and CRISPR Therapeutics in 2018 (CTX001) [41] and Allife Medical Science and Technology Co., Ltd. in 2019 (HBB HSC-01) [43], aim to treat patients with β-thalassemia and sickle-cell disease by the autologous transfusion of CRISPR/Cas9-edited CD34+ hematopoietic stem cells. Patients receiving CTX001 showed progress after a few months [44], which is indicative of the success of CRISPR application in gene therapy. EDIT-101, is being tested by Allergan and Editas Medicine since 2019 [45], to correct a *CEP290* splicing defect in patients of Leber Congenital Amaurosis, after showing significant therapeutic potential in a preclinical study [46]. These particular CRISPR therapies, and similar ones tackling hemoglobinopathies or eye diseases, entertain a key advantage over several others, listed below, which are “stuck” in their preclinical stages. This main advantage is the route of administration employed. Essentially, CTX001 and HBB HSC-01 involve ex vivo genome editing of cells which are transferred back to patients [41,43]. On the other hand, EDIT-101 is applied to patients intraocularly; a mode of delivery generally used for its local effects [45]. This circumvents the need for establishing a safe and efficient method of delivery that can reach the organ of interest within the human body, a major hurdle facing the translation of most CRISPR therapeutics to the clinic today.

To list a few examples of preclinical advances in CRISPR therapy, multiple groups have been successful in treating hereditary tyrosinemia I, using Cas9, in disease models by correcting disease-causing *FAH* mutations [47,48] or knocking-out *HPD* [49]. Similarly, researchers applied Cas9 to effectively correct a disease causing mutation in *CFTR* to treat monogenic cystic fibrosis in patient-derived induced pluripotent stem cells [50,51]. Cas13 was developed as an antiviral to target the genomes of RNA viruses such as Influenza and SARS-CoV-2 in human lung epithelial cells to combat airway diseases [52]. An ambitious approach towards modern airway disease pandemics with no current preventative vaccines or proven treatment, where the authors explain the necessity of finding an effective in vivo delivery method to the lungs [52]. Several promising strategies towards neurodegenerative diseases, currently with no cure, have also been reported. For example, using Cas9 to knock-out the expression of mutant amyloid precursor protein (APP) was demonstrated to have neuroprotective effects in a mouse model of Alzheimer’s disease [53]. One more notable approach utilizes Cas13 to convert the abundant astrocytes in the brain into dopaminergic neurons that are lost in Parkinson’s Disease in order to restore motor behavior in mice [54]. This latter example highlights the breadth of CRISPR’s therapeutic application especially because it tackles the disease using a cell-conversion strategy rather than editing a disease-causing mutation. Indeed, biomedical research has already developed various strategies for CRISPR therapy that can halt or reverse disease progression. However, the translation of such therapies is contingent upon the development of appropriate modes of delivery.

## 3. Limitations

The key challenges of translating CRISPR therapeutics to the clinic center around off-target effects, where unwanted genetic modifications occur, and the lack of efficient delivery systems that can specifically target CRISPR to the tissue of interest while avoiding undesirable gene editing in the remaining parts of the body. These limitations are addressed in current research by enhancing CRISPR’s gene recognition precision, and by developing and testing novel delivery vectors capable of tissue targeting.

### 3.1. Off-Targets

Off-target effects are non-specific genetic modifications that can occur when the CRISPR-Cas nuclease binds at a different genomic site than its intended target due to mismatch tolerance [17]. Cas9 sgRNA sequence contains 20nt which are complementary to the target DNA; however, it has been reported that tolerance of 3–5 mismatches distal to the PAM sequence can cause off-targets to occur [55]. Solutions such as a high fidelity Cas9 [56], paired nickases [57], dCas9-FokI nuclease fusion [58,59,60], and truncated gRNAs [61] for efficiently reducing off-target effects have been reported. A lower mismatch tolerance has been reported with Cas12a and is associated with a reportedly lower incidence of off-targets than native Cas9 [62].

Moreover, the chance of off-targets is increased with the prolonged exposure of the CRISPR-Cas complex to the genome, and strategies to limit this exposure time using ribonucleoproteins (RNPs) can lead to substantially lower off-targets than plasmid-based approaches [63]. With CRISPR-encoding plasmids, the continuous expression of CRISPR by cells over several days can result in a higher chance for off-target events [64].

### 3.2. Delivery

As previously discussed, a major impediment in the way of CRISPR-Cas translation to the clinic is the lack of an appropriate in vivo delivery carrier that can target CRISPR components to the cells of interest. Although, with the first clinical trial launched in 2018, this and subsequent CRISPR clinical trials rely on ex vivo gene editing of cells that are re-implanted back to the patient [41]. For many other diseases, the fulfilment of the CRISPR-Cas therapy is in its direct delivery into the patient’s body to treat their own cells directly, including cells which are difficult to be extracted and cultured outside the body. In order to reach this potential, researchers have developed several kinds of carriers such as viral vectors, and non-viral vectors such as lipids, peptides nanoparticles and others [65]. Allergan and Editas Medicine’s in vivo clinical trial involves the use of adeno-associated viruses (AAVs) [42], which are the most established delivery vectors in the field. However, due to safety and efficacy issues inherent to viral vectors, the development of safe non-viral vectors to deliver CRISPR-Cas components to target cells has gained much focus lately. The advantages of non-viral over viral vectors are discussed in Section 5.

## 4. Strategies for CRISPR-Cas Delivery

To carry out genome editing in vivo, CRISPR-Cas components, consisting of a Cas nuclease and a gRNA, must be efficiently delivered to target cells [66]. During delivery, CRISPR components are also exposed to degradation from serum and cellular proteases [67] and possibly to neutralizing antibodies [68]. Thus, a safe and efficient delivery system which can target a specific tissue or cell type, and can offer sufficient protection to CRISPR from degradation and immune responses is required. In later sections, we discuss progress made in CRISPR delivery systems, with a focus on non-viral vectors which offer several advantages over viral vectors such as safety, customizability and packaging capacity [69].

**Figure 3:**
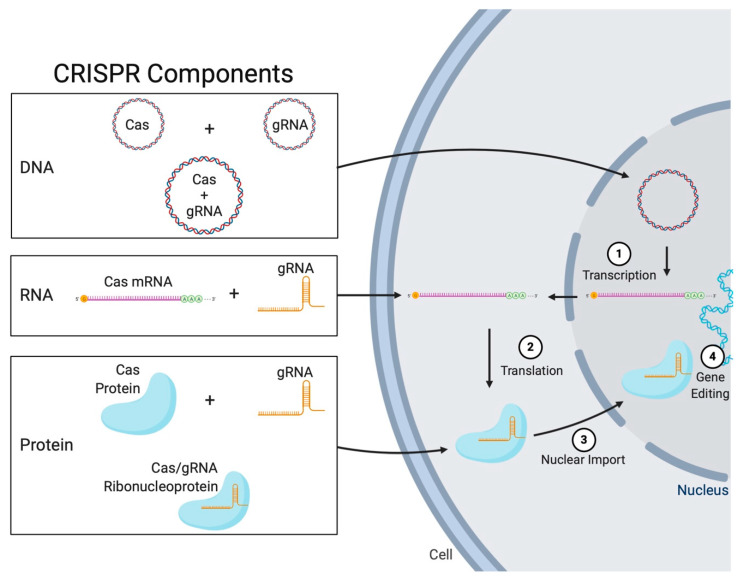
Different delivery platforms of Clustered Regularly Interspaced Short Pallindromic Repeats (CRISPR)-Cas system in human cells. Possible forms of CRISPR-Cas cargo to be delivered to cells (DNA, RNA, or protein) and intracellular processing: (1) a CRISPR-Cas encoding plasmid is transcribed to CRISPR-associated (Cas) messenger RNA (mRNA) and a guide RNA (gRNA) encoding plasmid is transcribed into a single-guide RNA (sgRNA). (2) mRNA moves to the cytoplasm to be translated into a Cas nuclease, (3) a sgRNA/Cas ribonucleoprotein complex (RNP) is imported into the nucleus. (4) gRNA/Cas RNP performs gene editing at the target site.

CRISPR-Cas nuclease, and gRNA can be delivered in several formulations (as illustrated in Figure 3):

1- DNA
  - Two plasmids; one encoding the protein and one encoding the gRNA [70].
  - A single plasmid encoding both components [71].
2- RNA
  - Cas protein encoded in a messenger RNA (mRNA) and the gRNA as an in vitro transcribed synthetic oligonucleotide [72].
3- Protein
  - Cas protein and a synthetic gRNA oligonucleotide [73].
  - Cas protein and gRNA RNP complex [74].

From the delivery point of view, using a single component is more efficient and practical because it does not require the design and co-loading of a separate delivery vector for each component [75]. Moreover, gene editing cannot occur without the presence of both components in the same cell (i.e., the chance for a cell to receive two components is less than receiving one component). For therapeutic purposes, using plasmids may have potential safety concerns because introduced DNA may interfere with the host genome causing insertional mutagenesis [76,77]. A general consensus is that it is preferable to deliver the Cas nuclease as a functional protein because it saves the time during which the plasmid needs to be expressed; naked gRNA is relatively unstable and may get diffused or degraded, becoming unavailable to form a RNP complex with the nuclease when protein expression is completed [64].

Ultimately, the goal of each strategy is the localization of a RNP complex inside the nucleus of host cells, and delivering it as a functional RNP complex is considered the most straightforward and efficient strategy, requiring the least amount of intracellular processing [64] (Figure 3). The RNP complex also significantly protects the delicate gRNA molecule from degradation, and it has a short half-life which reduces the chances for off-target mutations to occur [78]. Whereas, a CRISPR-encoding plasmid will continuously be expressed and may lead to undesired mutations [64]. Additionally, RNP delivery cannot trigger a cellular immune response associated with the presence of foreign plasmids inside cells [79]. RNP delivery also offers control over stoichiometry which is helpful in setting up dosage parameters in the development of therapy. Although delivering RNP complex is safer and more efficient, continuous plasmid expression may be more stable and is much less laborious to prepare. Indeed, plasmid DNA and RNP are the most widely used forms for CRISPR-Cas delivery (a comparison of strengths and weaknesses of each form is summarized in Table 2) [80,81].

## 5. Non-Viral Targeted Delivery Strategies for the CRISPR-Cas System

### 5.1. Advantages of Non-Viral over Viral Vectors

Most studies have relied on viral vectors that are known to cause immunogenic reactions in the host and carry the risk of insertional mutagenesis [82,83,84]. Although targeted delivery can be achieved via viral vectors, they may be more suited for application in cells for ex vivo therapy due to safety concerns [80,84]. An example of AAV-mediated targeted delivery, which are considered the safest of viral vectors, involves differential peptide display on the surface of viral particles which can influence the interaction between the viruses and different cell-types [85]. By screening a matrix of 12 AAV serotypes and 6 peptides, the team has developed an online superior peptide insertions for improved targeting (SPIRIT) database which contains data required to target different cell-types [85]. However, AAVs have a limited packaging size, and may lead to prolong expression of the delivered CRISPR-encoding plasmids thereby increasing off-target effects [80].

A clinically translatable cell-specific delivery method for CRISPR is consistently needed. Researchers have relied on techniques such as transfection reagents, electroporation, or micro-injection to deliver CRISPR components to cells, but these methods are limited to use in cells (in vitro/ex vivo) [86,87]. To overcome the challenges associated with the viral and physical delivery systems [84,88,89,90,91], several studies have been focused on developing non-viral delivery systems due to several advantages:

(1) Some synthetic molecules are unrecognizable by serum enzymes and can offer protection to the delivered components from degradation until they reach their target site [92,93].
(2) They are customizable and do not present a limited packaging capacity, which is compatible with the large-sized CRISPR components such as RNP or DNA.
(3) Unlike viral vectors, they do not cause mutagenicity or trigger an immunogenic response and achieve much higher reported safety profiles [83,94].
(4) They are composed of synthetic particles which can be engineered to bind cell-specific receptors, giving rise to disease-targeting avenues [95,96,97].
(5) They carry a very good scale-up potential, which greatly eases the process for clinical translation [98].

### 5.2. Non-Viral Vectors for Targeted Delivery of CRISPR

Non-viral delivery vectors for CRISPR used in research include liposomes, polymers, cell-penetrating peptides (CPPs), and other nanoparticles. For example, the ability of lipofectamine and other lipid-based carriers to complex and deliver CRISPR components has been reported [88]. Others have achieved delivery using DNA nanoclews [91] or gold nanoparticles [99]. Although some of these methods have achieved in vivo gene correction, most approaches do not offer cell-type specificity and are potentially toxic [66].

Generally, targeted delivery is based on cell-specific receptor-mediated recognition of ligands, triggering endocytosis in target cells. Studies demonstrating the targeted non-viral delivery of CRISPR have surged and continue to increase since 2017 (Table 3). The aim of targeting strategies is to increase the concentration of CRISPR at the target tissue and reduce undesirable gene editing in the rest of the body. In order to develop the CRISPR-Cas system as a clinical application, there are two main stages any method of delivery should facilitate. First, the delivery system should be able to specifically target CRISPR components into the cells of interest. Second, once CRISPR components are internalized, it is critical that they escape the denaturing environment of the endosome, as will be addressed later in the review, in order to gain access to the nucleus and perform gene editing.

Targeting asialoglycoprotein receptors (ASGPrs), such as galactose receptors expressed on hepatocytes, represent an attractive strategy for liver-specific CRISPR therapies treating conditions such as hepatic cancer and hypercholesterolemia [101,103,113]. An example of this was demonstrated in the laboratory of the CRISPR pioneer Jennifer A. Doudna, showing that chemo-selective ligands, based on variations of galactosamine, were covalently fused to solvent-exposed cysteines of a mutant version of Cas9 by di-sulfide bond formation [113]. The study showed evidence of liver cell-specific internalization and successful editing of the *EMX1* gene in a human hepatocarcinoma cell line [113]. However, their strategy required the complex engineering of Cas9 protein and is only applicable to cells. They do not provide means for protection from enzymatic degradation, and relied on the co-addition of endosomolytic peptides to promote endosomal escape [111,113,114]. A second example of liver-specific delivery was shown by incorporating Cas12 RNPs into DNA nanoclews, coated with a galactose-conjugated cationic polyethyleneimine polymer. Interestingly, this team additionally incorporated an anionic so-called “charge reversal” layer, which turns cationic in the acidic conditions it is exposed to in the endosome [103]. This promotes a proton-sponge effect to take place and ruptures endosomal vesicles to release RNPs to the cytosol. Using this strategy, the team were able to disrupt *PCSK9* and restore cholesterol levels in mice [103]. Targeting a Cas9-encoding plasmid to liver-specific ASGPr receptors was also achieved using a lactose-functionalized biopolymer to knock-out *survivin* gene in hepatocarcinoma mouse models [101]. Similarly, CRISPR-encoding mRNAs were targeted to knock-out *HPV18E6* and stop the growth of cancer cells using lipid nanoparticles functionalized with phenylboronic acid, which interacts with cancer cellular surface sialic acid [107].

Many targeting approaches rely on cell-specific aptamers because they can be relatively easily synthesized, and screened against a wide range of targets. DNA aptamers offer several unique advantages such as cost-effectiveness, non-immunogenicity, and reduced non-specific binding to other cell-types due to their net negative charge [100]. For example, DNA aptamer-decorated extracellular vesicles loaded with Cas9 RNPs achieved tumor-specific targeting and *WNT10B* knock-out in human primary liver cancer-derived organoids and xenograft tumor models, and subsequent tumor growth inhibition in vivo [100]. Others were able to deliver functional Cas9 RNPs through a complex mixture of particles including a targeting AS1411 aptamer to successfully up-regulate the PI3K/AKT pathway and trigger apoptosis in tumorous cells [105,108]. AS1411 is a 26-bp DNA oligonucleotide that can interact with nucleolin which is abundantly expressed on the surface of cancer cells [105,108]. The targeted delivery of CRISPR/Cas9-encoding plasmids was also demonstrated in many cases by tissue-specific RNA, DNA, and peptide aptamer-functionalized liposomes to target and treat osteosarcoma, pancreatic, prostate and brain cancers in mice [109,115,117].

Remarkable work was seen in the development of a selective organ targeting (SORT) system which is compatible with CRISPR-Cas mRNA/gRNA as well as RNP complexes [104]. This strategy is based on altering the internal charge of lipid nanoparticle carriers by modifying their molar compositions of an additional “SORT” molecule to change their cell-fate [104]. Using this technique, the research team has shown evidence for lung, liver and spleen specific targeting in mice, and efficient gene editing of *PTEN* and *PCSK9*, therapeutic targets for cancer and hypercholesterolaemia, respectively [104]. Similarly, Wei and colleagues achieved liver and lung-specific delivery of lipid nanoparticle-encapsulated RNPs in mice [102]. Tissue-specific targeting was also achieved by adjusting the lipid nanoparticles molecular composition and ratios [102]. In order to disrupt the endosomal membrane, they used ionizable lipid nanoparticles which can acquire charge once inside the endosome acidic environment [102]. This strategy was used to create organ-specific cancer mouse models by targeting multiple genes in the livers and lungs, and to significantly decrease serum *PCSK9* levels in mice [102]. Moreover, a noteworthy approach exploiting the homotypic binding phenomenon of tumor cells involved coating nanoparticles harboring Cas9 RNPs with MCF-7 cancer cell membranes to bind and target homotypic cancer cells [106].

All the above methods involve the tailoring and multistep synthesis of complex particles, the translation of which may be impractical for clinical practice. Importantly, they primarily rely on cell-specific receptor recognition, for targeting the delivery of CRISPR-Cas components to target cells.

## 6. The Evolution of Peptide Delivery Systems for CRISPR-Cas Components

Peptide-mediated delivery of a Cas9 protein and gRNA to cells was first reported by Ramakrishna and colleagues [90], with gene-editing efficiencies comparable to plasmid transfection, as measured using a surrogate reporter system that can detect Cas9-induced mutations [84,90,118]. The first report of a peptide-mediated cell-targeted delivery of CRISPR/Cas9 RNP in vivo was through the fusion of a P2C yolk protein fragment to Cas9 to promote mosquito oocyte-specific penetration [114]. This technique relied on the co-treatment of endosomal escape reagents to achieve gene editing [114]. However, the above approaches involve the covalent fusion of Cas9 to a CPP, which is considered to “hold back” the endonuclease from reaching its target to perform gene editing, rendering it relatively inefficient [73,90,119]. Therefore, harsh treatment conditions involving a series of incubations are required in order to reach levels of gene editing comparable to established methods. This “crippling” effect has also been reported in a study delivering a parathyroid hormone conjugated to a CPP [120].

The strategy of peptide-mediated delivery of CRISPR-Cas systems was later evolved to address this problem [119]. Essentially, it was demonstrated that CRISPR RNPs can be readily and non-covalently assembled within cationic peptides through simple incubation, and form nanoparticles capable of effectively penetrating the cell membrane of cells, similar to conventional lipofection protocols [119]. This was confirmed in several cell types achieving levels of gene editing comparable to Lipofectamine 2000 and with less cytotoxicity [119].

**Figure 4:**
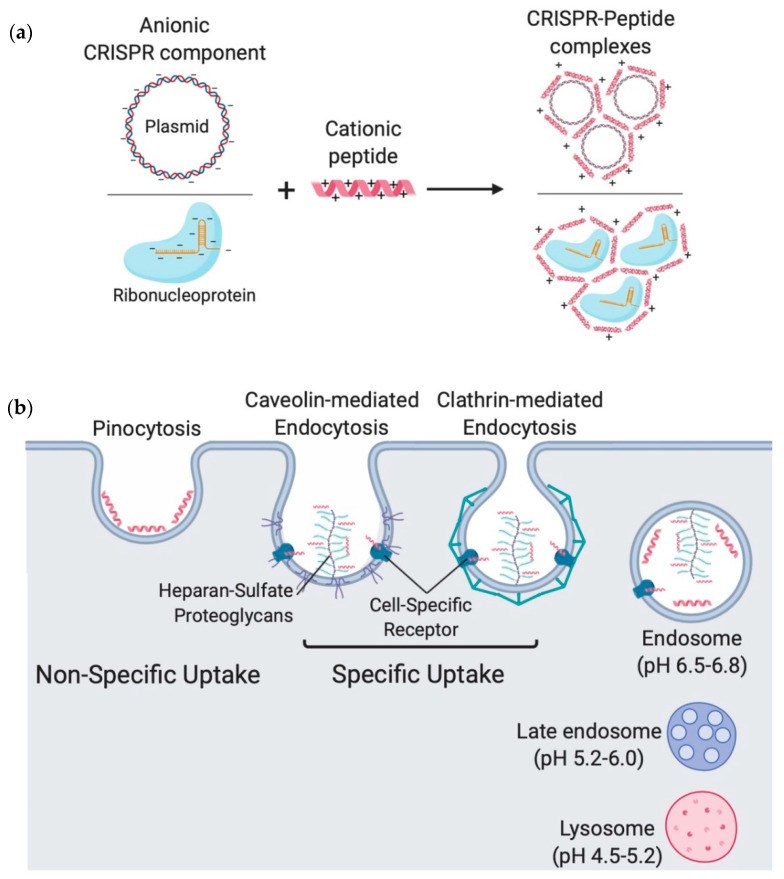
Mechanism of Clustered Regularly Interspaced Short Pallindromic Repeats (CRISPR)–peptide complexes formation and cellular uptake. (**a**) An anionic ribonucleoprotein or CRISPR-encoding plasmid forms a complex with cationic peptides through charge–charge interactions. The negatively charged phosphate groups of nucleic acids found on guide RNA (gRNA) and DNA molecules form salt bridges with the positively charged amino acids of cationic peptides. (**b**) Cell-penetrating peptides (CPPs) can be internalized into cells non-specifically through pinocytosis or specifically through cell-specific receptor-mediated endocytosis which can be caveolin or clathrin-dependent. Internalized CPPs are localized within endosomes where it is crucial that their cargo escapes into the cytoplasm before the lysosomal stage to avoid loss of integrity due to exposure to the acidic environment or enzymatic degradation.

Cationic CPPs form complexes with CRISPR components through charge–charge interactions (Figure 4A); Cas9 protein (theoretical net charge +22) complexed with gRNA (theoretical net charge −101) forms an anionic RNP complex with a theoretical net charge of −79. Theoretically, electrostatic interactions are initially formed between the negatively charged phosphate groups of nucleic acids in gRNA and the positively charged side chains of amino acids on peptides, and are further stabilized by hydrogen bonds during self-assembly. The interplay between these molecular interactions results in the formation of salt bridges; reminiscent of the mechanism through which histones are bound to DNA in the nucleus [121,122]. In the same manner, a CRISPR-encoding plasmid can also form complexes with cationic peptides; CPPs have been extensively used for the delivery of nucleic acids, which is well presented in detail in the literature [123,124].

**Figure 5:**
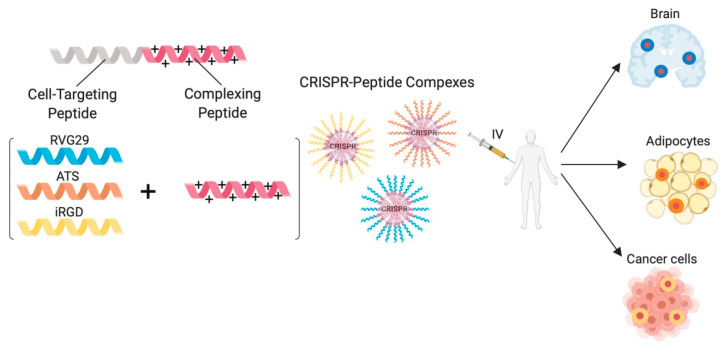
A possible universal peptide-based approach for targeting the delivery of CRISPR components to different cell-types. Cell-specific targeting peptides are composed of a cell-targeting peptide, essential for cell-specific receptor interaction, fused to a cationic complexing peptide required for binding anionic CRISPR components. CRISPR-peptide complexes can be administered intravenously to reach their target tissue/organ. (RVG29: rabies virus glycoprotein 29; ATS: adipose targeting sequence; iRGD: integrin-specific arginylglycylaspartic peptide).

Peptides have long been used to achieve tissue-specific delivery of a variety of cargoes, such as drugs, nucleic acids and nanoparticles [125]. To achieve tissue-specific delivery of CRISPR components, a few approaches further involved peptides containing a targeting moiety. For example, Chung et al. achieved targeted delivery of a CRISPR-encoding plasmid via complexing with a cyclized peptide combining a short adipose-targeting peptide and a cationic D-form nona-arginine (ATS-9R) by charge–charge interactions [96]. ATS binds prohibitin expressed on adipocytes and achieved preferential uptake in vivo, efficiently knocking down *Fabp4* gene implicated in obesity [96]. Later, the targeted delivery of a CRISPR/Cas9 RNP to knock-out *CD71* over-expressed in cancer cells was reported [110]. This was achieved through a peptide combining a cationic CPP for complexing and a targeting integrin-specific arginylglycylaspartic acid (iRGD) peptide that can interact with αvβ3 integrins found on cancer cells [110]. The iRGD peptide is a tumor homing motif routinely used for cancer targeted delivery of drugs, biologics and nanoparticles [126]. Using amphiphilic peptides, another study established the delivery of CRISPR/Cas9 and Cas12 RNPs to airway epithelial cells in mice [112]. A strategy deemed promising for delivering Cas13 complexes to combat airway diseases as previously mentioned in this review [52]. However, the latter studies do not offer much evidence of preferential uptake as only the cells of interest were used in vitro, and urgently need to generate sufficient data from in vivo experiments. Nonetheless, the progress made represents huge promise for the development of peptide-mediated tissue-targeting CRISPR therapies in the imminent future (Figure 5).

## 7. CPPs

CPPs are short stretches of amino acids (5–30 amino acids in length), usually cationic and basic, that are used as vectors for delivery due to their cell-penetrating potential. The first CPPs discovered are the trans-activator of transcription (TAT) protein of the human immunodeficiency virus (GRKKKRRQRRRPQ) [127], and the antennapedia homeodomain of *Drosophila* (penetratin: RQIKIWFQNRRMKWKK) [128]. Discovering new CPPs, and understanding their mechanism soon became the focus of intense research. Now, around 2000 natural and synthetic CPPs have been reported [129]. Although, their mechanism is a subject of debate, CPPs have been used to deliver a range of cargoes, including proteins and nucleic acids, in vitro and in vivo [130].

CPPs offer advantages over other non-viral vectors in terms of efficacy, specificity and safety, especially since they are naturally occurring biologics. Relatively, peptides offer a great chemical diversity, as each residue carries a functional group which can further be altered chemically giving rise to further diversity. Many peptides are inspired from naturally occurring proteins and thus carry biological activity and can interact with membrane proteins and receptors [131]. Unlike synthetic molecules, proteins and peptides regulate virtually all physiological processes, and when degraded into amino acids they do not produce toxic metabolites that can lead to an immunogenic response [132,133]. Considering their simple structure and makeup, they are relatively easy and less costly to manufacture with today’s technology [132]. In comparison, other molecules may require more complex design and lead to the production of toxic metabolites which can cause undesired interactions in vivo [132].

The disadvantages of peptides include susceptibility to degradation by proteolytic enzymes, and non-specific interactions with different cell receptors due to a high structural flexibility. However, advancements in peptide design have led to the ability to synthesize peptides with improved properties. For example, increased peptide stability has been achieved using d-amino acids, which are unrecognized by serum and cellular proteases, unlike the naturally-occurring l-amino acids [134]. Moreover, it was also shown that the incorporation of cysteines at particular sites within a peptide leads to di-sulfide bond formation and grants the peptide a rigid structure, greatly improving its activity, specificity and stability [135]. For example, cyclic CPPs retain a number of advantages over linear CPPs, including a superior membrane permeability, resistance to proteolytic degradation, enhanced endosomal escape, and improved affinity [136]. Their restrained conformational freedom largely improves their activity by exposing their interactive functional groups, and by reducing entropy loss and the energy required for binding [137,138]. For these reasons, peptides have become attractive in drug design and treatment of different diseases [132,133,139].

## 8. Mechanism of CPP-Uptake and Release

### 8.1. Cellular Uptake

It is not yet fully understood how CPPs are taken up by cells, but there is a general consensus that this takes place through different kinds of endocytoses [140] (Figure 4B). It is important that a CPP associates with the plasma membrane in order to either enter a cell along with a physiological endocytic event, or to actively trigger endocytosis. CPPs can bind to various structures on the cell membrane such as phospholipids, proteoglycans or cell-surface receptors to promote uptake processes such as pinocytosis, clathrin-mediated and caveolin-mediated endocytoses [141,142,143,144,145] (Figure 4B).

Charge–charge interactions are key to initiate the first contact between peptides and the cell membrane. Hydrogen bonding then occurs between amino acid side chains and phosphate groups of membrane phospholipids or sulfate groups of heparan-sulfate proteoglycans (HSPGs) [146,147,148,149,150]. Apart from charge–charge interactions, amphipathic CPPs containing hydrophobic residues (such as RW9: RRWWRRWRR) were shown to partake in hydrophobic interactions with membrane phospholipids [151,152]. Importantly, cell-surface receptors have been shown to engage in sequence-specific uptake of CPPs. For example, neuronal nicotinic acetylcholine receptors which are known to bind rabies virus glyocoprotein 29 (RVG29) peptide [131,153], cancer-expressed integrins that bind iRGD peptide [110,126], and prohibitins on adipocytes that bind ATS [96,154].

### 8.2. Endosomal Release

After endocytosis, internalized material is found in endosomes (pH 6.5–6.8), which develop to be late endosomes (pH 5.2–6.0), before moving into lysosomes (pH 4.5–5.2) where it is subjected to degradation by enzymes and the acidic environment (Figure 4B) [155]. Endosomal escape is considered to be the bottleneck in the development of most non-viral vectors, including CPPs, with very low endosomal escape efficiencies [155,156]. This has been reported, in many studies employing CPPs to deliver plasmids, proteins or siRNA, as punctate distribution of the internalized CPPs, suggesting that they are remaining within endosomal vesicles [157,158,159].

The mechanism of endosomal escape remains poorly understood, partially because of the lack of efficient endosomal release systems, however, some hypotheses have been proposed [160]. The “Proton Sponge” effect relies on the buffering capacity of polyamines at lysosomal pH which absorb hydrogen molecules into the endosome, eventually leading to its osmotic swelling and rupture, releasing the cargo [161]. Alternatively, molecules delivered using liposome-based methods escape the endosome through “Membrane Fusion” [162]. However, CPPs do not undergo either of these pathways because (1) they are basic and do not have a buffering capacity at physiological pH, and (2) they are not engulfed within liposomes. It has been proposed that CPPs may escape the endosome by forming “transient pores” in the endosomal membrane through which they can passively diffuse [163]. A mechanism of “local membrane disruption” has been proposed for small molecules associated with CPPs to ‘leak’ through the endosome without completely destroying it [164]. Lastly, a “vesicle budding and collapse” mechanism explains that CPPs gradually cluster in the intraluminal endosomal membrane within rafts where they can eventually bud off as vesicles that disintegrate into the cytoplasm [165].

With the CRISPR-Cas systems, especially large sized genes (~4.2 Kbp for the most commonly used SpCas9) or proteins (~100–160kDa) [80], endosomal escape may present a real problem. For this reason, researchers usually introduce endosomolytic agents (such as chloroquine, Ca^2+^, and endosomolytic peptides) to enhance endosomal escape [105,111,113,114]. Co-treating cells with ppTG21 peptides was shown to promote the endosomal escape of CRISPR proteins [111,113] (Table 3). Alternatively, using cyclic CPPs is reportedly effective in tackling endosomal escape due to their improved membrane permeability [150,166,167,168]. For example, the cyclic ATS-9R was used to deliver CRISPR-encoding plasmids with comparable efficiency to lipofectamine [96]. Moreover, amphiphilic peptides have been used due to their partial hydrophobicity which can interact with and disrupt lipid bilayer endosomal membranes [110,119,169,170,171,172]. For example, CPPs with incorporated lipid tails were employed for the delivery of CRISPR RNPs to cells, resulting in gene editing efficiencies comparable to commercial liposomal transfection reagents [110,119]. Similarly, the amphiphilic _6_His-CM18-PTD4 peptide (6His tag, PTD4: YARAAAARQARA, CM18: KWKLFKKIGAVLKVLTTG), which is known to escape endosomes through vesicle budding and collapse [173], was used to deliver CRISPR RNPs into cells [171]. Also, to circumvent the lysosomal pathway, pardaxin-decorated liposomes delivered a CRISPR/Cas9-encoding plasmid with higher efficiency than lipofectamine [172]. Due to the possible cytotoxicity of these membrane disruptive peptides, amphiphilic peptides which are only activated at the acidic environment of endosomes are used [174]. An example of these so-called “fusogenic pH-responsive” peptides is HA2 (GLFGAIAGFIENGWEGMIDGWYG), derived from the hemagglutinin influenza virus glycoprotein, which is used to enhance gene delivery [169,170]. Aspartate and glutamate residues are protonated at low pH, increasing the peptide hydrophobicity and resulting in interaction with and disruption of the endosomal lipid bilayer [175].

## 9. Conclusions

Although the field of CRISPR therapy has flourished during the last decade, there remains a need for developing appropriate targeted delivery systems to advance its use to the clinic. Developing non-viral delivery systems for CRISPR have been a subject of immense research to avoid the safety concerns associated with viral vectors. Thus far, most targeted delivery systems employed for CRISPR-Cas are dependent on ligand-decorated liposomes or nanoparticles, which require complex design or may be toxic to achieve cell-specific receptor recognition. Peptide-based non-viral delivery systems contain properties that offer advantages such as chemical diversity, low toxicity, resistance to proteolysis and ability for specific targeting. The non-covalent complexing of CRISPR-Cas components with peptides through charge–charge interactions represents a one-step, simple, safe, translatable, and customizable method for specific tissue targeting. Targeting CRISPR-Cas systems to the cells of interest carries a great deal of hope for biomedical research and for achieving the safety levels required for clinical translation. More research is needed to test the efficacy of peptide-based systems for the targeted in vivo delivery of CRISPR-Cas to different tissues.

## Figures and Tables

**Table 1:** Class 2 CRISPR systems which employed for therapeutic genome editing.

Class	Type	Nuclease	gRNA Structure			Target			Cleavage
			crRNA	tracrRNA	gRNA Length	Molecule	PAM	Availability in Human Genome	
2	2	Cas9	20nt (complementary to target)	85nt	105nt	dsDNA	5′-NGG (SpCas9)	Every ~8 bp	Blunt-ended DSB
	5	Cas12	40nt (20-24nt complementary to target)	None	40nt	dsDNA	5′-TTTN	Every ~23 bp	Sticky-ended DSB
	6	Cas13	64nt (23-30nt complementary to target)	None	64nt	ssRNA	None	Any location	Arbitrary cleavage around target site

Abbreviations: gRNA (guide-RNA), crRNA (CRISPR RNA), tracrRNA (trans-activating RNA), PAM (protospacer adjacent motif), Cas9 (CRISPR-associated protein 9), nt (nucleotide), bp (base pair), dsDNA (double-stranded DNA), SpCas9 (*Streptococcus pyogenes* Cas9), DSB (double-stranded break), Cas12 (CRISPR-associated protein 12), Cas13 (CRISPR-associated protein 13), ssRNA (single-stranded RNA).

**Table 2:** A features comparison between the different modes of delivery.

Feature	DNA	RNA	Protein
Cost	++	+	+++
Stability	+++	+	++
Editing Efficiency	+	++	+++
Rapidity	+	++	+++
Insertional Mutagenesis	+	−	−
Immunogenicity	+++	++	+
Off-targets	+++	++	+
Duration in cells	+++	++	+

The symbols (−, +, ++ and +++) mean none, low, moderate and high respectively.

**Table 3:** A summary of recent approaches of targeted delivery of CRISPR-Cas systems.

Carrier	Molecule	Target	Model	Disease/Gene	Reference
Valency-controlled tetrahedral DNA nanostructures conjugated with DNA aptamer	RNP	Tumor cells	In vivo	Cancer: *WNT10B*	Zhuang et al. 2020 [100]
Lactose-derived branched cationic biopolymer	Plasmid	ASGPrs on Liver cells	In vivo	Hepatocellular carcinoma: *survivin*	Qi et al. 2020 [101]
Classic Lipid Nanoparticles supplemented with permanently cationic lipid formulations	RNP	Lung, Liver	In vivo	Hypercholesterolemia: *PCSK9*, Lung Cancer: *PTEN*	Wei et al. 2020 [102]
Galactose-functionalized polyethyleneimine-coated DNA nanoclews	RNP	ASGPrs on Liver cells	In vivo	Hypercholesterolemia: *PCSK9*	Sun et al. 2020 [103]
Lipid Nanoparticles with SORT Supplemental molecules	mRNA/gRNA RNP	Liver, Lungs, Spleen	In vivo	Cardiovascular Disease: *PCSK9*	Cheng et al. 2020 [104]
Functionalized carrier: - Ca^2+^, Protamine, CO_3_^2−^, Hyaluronic acid chain, - Targeting Aptamer (AS1411), - Cell-Penetrating Peptide (TAT-NLS)	Plasmid	CD44 receptors on tumor cells	In vitro	Cancer: *CTNNB1*	He et al. 2020 [105]
Metal Organic Frameworks (Zeolitic imidazolate) encapsulating CRISPR/Cas9, coated with MCF-7 cancer cell membrane	RNP	Antigens on adenocarcinoma cells	In vivo	*EGFP*	Alyami et al. 2020 [106]
Phenylboronic acid-functionalized Lipid Nanoparticles	mRNA	Cellular surface sialic acid on cancer cells	In vitro	Cancer: *HPV18E6*	Tang et al. 2019 [107]
Cyclic ATS-9R Peptide: CKGGRAKD-rrrrrrrrrC	Plasmid	Prohibitin on adipocytes	In vivo	Diabetes: *Fabp4*	Chung et al. 2019 [96]
Functionalized carrier: - Ca^2+^, Protamine, CO_3_^2-^, Alginate chain, - Targeting Aptamer (AS1411), - Cell-Penetrating Peptide (NLS)	Plasmid	AS1411 receptors on tumor cells	In vitro	Cancer: *PTK2*	Liu et al. 2019 [108]
Liposome functionalized with R8-dGR peptide: Cys-RRRRRRRRdGR	Plasmid	NRP-1 and integrin αvβ3 on tumor cells	In vivo	Pancreatic cancer: *HIF-1α*	Li et al. 2019 [109]
CRISPR-GPS: - Targeting Peptide: Cyclic iRGD: CRGDKGPDC - Cell-Penetrating Peptide (mTP): GWTLNSAGYLLGKINLKALAALAKKIL	RNP	αvβ3 integrins on cancer cells	In vitro	Cancer: *CD71*	Jain et al. 2019 [110]
- IL-31 or NGF SNAP-ligands, - Cationic Protamine Peptides, - Endosomolytic Peptide (ppTG21)	Protein	IL-31 and NGF receptors on keratinocytes	In vivo	Skin disease: *Atat1*	Maffei et al. 2019 [111]
- S10 Peptide: KWKLARAFARAIKKLGGSGGGSYARALRRQARTG	RNP	Airway Epithelia	In vivo	Airway diseases: *CFTR*, *HPRT1*	Krishnamurthy et al. 2019 [112]
- Asialoglycoprotein receptor ligands, - Endosomolytic Peptide (ppTG21): GLFHALLHLLHSLWHLLLHA	RNP	ASGPrs on liver cells	In vitro	*EMX1*	Rouet et al. 2018 [113]
Recombinant Cas9 protein fused to Targeting Peptide: P2C on *C*-terminus	RNP	Yolk Protein receptors on mosquito oocytes	In vivo	*kmo*	Chaverra-Rodriguez et al. 2018 [114]
ssDNA LC09-functionalized PPC lipopolymer	Plasmids	Osteosarcoma	In vitro	Osteosarcoma: *VEGFA*	Liang et al. 2017 [115]
RNA aptamer A10-functionalized liposome	gRNA	Prostate-specific membrane antigen on cancer cells	In vivo	Prostate Cancer: *PLK1*	Zhen et al. 2017 [116]

Abbreviations: *WNT10B* (Wnt Family Member 10B); ASGPr (Asialoglycoprotein receptor); SORT (Selective ORgan Targeting), *PCSK9* (Proprotein Convertase Subtilisin/Kexin Type 9), *PTEN* (Phosphatase and tensin homolog), TAT (trans-activator of transcription), *CTNNB1* (β-catenin), *EGFP* (Enhanced Green Fluorescent Protein reporter gene), *HPV18E6* (Human Papillomavirus Type 18 E6), ATS (Adipocyte Targeting Sequence), 9R (nona-arginine), *Fabp4* (fatty acid binding protein), *PTK2* (protein tyrosine kinase 2), *HIF-1α* (hypoxia-inducible factor-1α), iRGD (integrin-specific arginylglycylaspartic acid peptide); GPS (Guiding Peptide Sequence), mTP (“m” = myrisotyl group, “TP” = transportan), *CD71* (transferrin receptor), *Atat1* (Alpha Tubulin Acetyltransferase 1), S10 (Shuttle10), *CFTR* (Cystic fibrosis transmembrane conductance regulator), *HPRT1* (Hypoxanthine Phosphoribosyltransferase 1), *EMX1* (Empty Spiracles Homeobox 1), *kmo* (kynurenine monooxygenase), *VEGFA* (Vascular endothelial growth factor A), *PLK1* (polo-like kinase 1).

## Abbreviations

9R	Nona-arginine
AAV	Adeno-Associated Virus
ASGPr	Asialoglycoprotein receptor
ATS	Adipocyte Targeting Sequence
bp	Base pair
Cas	CRISPR-associated
CPP	Cell-penetrating peptides
CRISPR	Clustered Regularly Interspaced Short Palindromic Repeats
crRNA	CRISPR RNAs
dCas9	Deficient Cas9
DNA	De-oxyribonucleic acid
DSB	Double-stranded break
gRNA	Guide RNA
HDR	Homology-Directed Repair
HSPG	Heparan sulfate proteoglycans
mRNA	Messenger RNA
NHEJ	Non-Homologous End Joining
PAM	Protospacer adjacent motif
RNA	Ribonucleic acid
RNP	Ribonucleoproteins
RVG	Rabies Virus Glycoprotein
sgRNA	Single guide RNA
SORT	Selective ORgan Targeting
SPIRIT	Superior Peptide InseRtions for Improved Targeting
TAT	Trans-activator of transcription
tracrRNA	Trans-activating RNA
